# 4089 Clinical Implementation of Monte Carlo Dose Calculation for Patient-Specific Radiotherapy Quality Assurance

**DOI:** 10.1017/cts.2020.327

**Published:** 2020-07-29

**Authors:** Holly Marie Parenica, Christopher Kabat, Pamela Myers, Neil Kirby, Pavlos Papaconstadopoulos, Nikos Papanikolaou, Sotirios Stathakis

**Affiliations:** 1 University of Texas Health Science Center San Antonio; 2 Netherlands Cancer Institute

## Abstract

OBJECTIVES/GOALS: The Monte Carlo dose calculation method is often considered the “gold standard” for patient dose calculations and can be as radiation dose measurements. Our study aims to develop a true Monte Carlo model that can be implemented in our clinic as part of our routine patient-specific quality assurance. METHODS/STUDY POPULATION: We have configured and validated a model of one of our linear accelerators used for radiation therapy treatments using the EGSnrc Monte Carlo simulation software. Measured dosimetric data was obtained from the linear accelerator and was used as the standard to compare the doses calculated with our model in EGSnrc. We will compare dose calculations between commercial treatment planning systems, the EGSnrc Monte Carlo model, and patient-specific measurements. We will implement the Monte Carlo model in our clinic for routine second-checks of patient plans, and to recalculate plans delivered to patients using machine log files. RESULTS/ANTICIPATED RESULTS: Our Monte Carlo model is within 1% agreement with our measured dosimetric data, and is an accurate representation of our linear accelerators used for patient treatments. With this high level of accuracy, we have begun simulating more complex patient treatment geometries, and expect the level of accuracy to be within 1% of measured data. We believe the Monte Carlo calculation based on machine log files will correlate with patient-specific QA analysis and results. The Monte Carlo model will be a useful tool in improving our patient-specific quality assurance protocol and can be utilized in further research. DISCUSSION/SIGNIFICANCE OF IMPACT: This work can be implemented directly in clinical practice to ensure patient doses are calculated as accurately as possible. These methods can be used by clinics who do not have access to more advanced dose calculation software, ensuring accuracy for all patients undergoing radiotherapy treatments.

